# Aerobic Exercise Combination Intervention to Improve Physical Performance Among the Elderly: A Systematic Review

**DOI:** 10.3389/fphys.2021.798068

**Published:** 2022-01-04

**Authors:** Xiaorong Bai, Kim Geok Soh, Roxana Dev Omar Dev, Othman Talib, Wensheng Xiao, Kim Lam Soh, Swee Leong Ong, Chenyang Zhao, Ovidiu Galeru, Catalina Casaru

**Affiliations:** ^1^Department of Sports Studies, Faculty of Educational Studies, Universiti Putra Malaysia, Seri Kembangan, Malaysia; ^2^Department of Science and Technical Education, Faculty of Educational Studies, Universiti Putra Malaysia, Serdang, Malaysia; ^3^Department of Nursing, Faculty of Medicine and Health Sciences, Universiti Putra Malaysia, Seri Kembangan, Malaysia; ^4^School of Nursing Science, Faculty of Medicine, Universiti Sultan Zainal Abidin, Terengganu, Malaysia; ^5^Human Resources Office, Wuxi Vocational Institute of Arts and Technology, Wuxi, China; ^6^Faculty of Movement, Sports, and Health Sciences, “Vasile Alecsandri” University of Bacau, Bacǎu, Romania; ^7^Department of Physical Education and Athletic Training, University of West Alabama, Livingston, CA, United States

**Keywords:** strength training, flexibility, balance, gait, body composition

## Abstract

The benefits of aerobic exercise for the elderly are well-known. They extend beyond cardiovascular changes and can reduce the inactivity-induced loss of strength, mobility, balance, and endurance that are vital for the safe performance of daily activities in older adults. However, the benefits of combined aerobic exercise with other exercises such as strength/resistance, multi-component and aerobic exercise remain unknown. The purpose of this study is to examine the effects of combined aerobic exercise on physical performance among the elderly, as opposed to single aerobic exercise. We searched four databases of SCOPUS, PubMed, EBSCOhost, and CINAHL Plus to find 18 articles that met criteria. Data was extracted using PICOs extraction tool and summarized using a narrative synthesis approach. Studies have shown that aerobics combined resistance/strength training (CEX), multi-component training (ME), and dance combined training has positive and significant effects on the physical performance (upper body strength and lower body strength, dynamic balance, fall risk, mobility, gait, agility, flexibility) of the elderly. CEX had additional benefits compared to aerobic training (AER) and resistance/strength training (RES) in gait speed, lower limb strength, and trunk fat. Furthermore, CEX was more effective than AER in improving sitting and stretching, elbow flexion, knee flexion, shoulder flexion and stretching, strength and body fat, function reach test, 30-s chair standing test and 6-min walking test, self-evaluation of body function. Therefore, the combination of multiple components contributes to the overall improvement in physical fitness of the elderly, thus preventing them from losing balance and reducing susceptibility to injury.

**Clinical Trial Registration:** [https://www.crd.york.ac.uk/prospero/#recordDetails], identifier [CRD42021213147].

## Introduction

According to the National Commission on aging, about 77% of the elderly have at least two chronic disease (NCOAging, 2021). Heart disease, stroke, cancer and diabetes are the most common and expensive chronic diseases causing two-thirds of deaths each year (Ross, 2019). According to statistics, 50% of the naturally aging over the age of 50 suffer from osteoarthritis, and 90% of people over 65 suffer from this disease (Xinhuanet, 2017). Falls are the world’s second leading cause of accidental death after traffic accidents, and the main cause of personal injury, especially for the elderly (Ambrose et al., 2013).

Faced with the health threats of multiple diseases and falls, a study released by the World Health Organization (Kyu et al., 2016) recommends approximately 150 min of moderate-intensity physical activity or exercises per week, which can reduce the risk of common chronic illnesses such as breast cancer, type 2 diabetes and stroke (Hojman et al., 2018) besides boosting the immunity (Simpson et al., 2015). Aerobic exercise as a form of physical activity. Aerobic exercises are the ones that focus on pumping the oxygenated blood from heart to other working muscles. Any exercise that pumps blood to the larger and smaller working muscles can be considered as an aerobic exercise. One of the main benefits of aerobic exercise is the enhancement of cardiovascular health (Bakken et al., 2001). It has shown that aerobic exercise can reduce the inactivity-induced loss of strength, mobility, balance, and endurance vital for the safe performance of daily activities in the elderly (Bakken et al., 2001; Fragala et al., 2019). However, continuous efforts to identify and refine physical performance are critical in improving public health (Sousa et al., 2014). Most people tend to focus on one type of exercise or activity, and perceive they are exercising enough. However, the pertinence of different exercises varies. For example, the balance exercise component is better than the aerobic exercise component in terms of preventing falls (Rose, 2008). The flexibility component exercise is better for arthritis (Suomi and Collier, 2003). Strength training can delay aging, and endurance training has a better effect on Non-communicable diseases (NCDs) (Batmyagmar et al., 2019). Applying the benefits of different exercise components for the elderly to prevent the crisis of multiple diseases and accidental injuries still needs to be explored.

The national Institute on Aging (NCOAging, 2021) reports that it is vital to get all four types of exercise: endurance, strength, balance, and flexibility. It also advocates the combination of various exercise components to comprehensively promote the physical health of the elderly and prevents injuries caused by the imbalances between the enhancement of one component and neglecting the other (Woo et al., 2007; Timmons et al., 2018). Moreover, there are many combinations of aerobic exercises that have shown more benefits from the combination of multiple exercise components than from one component alone (Rogers et al., 2002; Deley et al., 2007; Zhuang et al., 2014; Lee et al., 2015; Lacroix et al., 2016; Haripriya et al., 2018; Jia et al., 2018; Timmons et al., 2018), and the effect of the combination of multiple exercise components is greater than the effect of one component exercise (Cancela Carral and Ayán Pérez, 2008; Sousa et al., 2013a, 2017; Lee et al., 2015; Timmons et al., 2018; Lichtenstein et al., 2020). The effects of combination workouts vary with the type, duration, and frequency of exercise. American College of Sports Medicine (ACSM) Exercise for older adults (Chodzko-Zajko, 2014) pointed out that the type of physical activity that is suitable for the elderly varies from person to person. It is recommended that the elderly select several favorite sports and combine them to boost their physical strength, endurance, flexibility, and balance. At the present stage, there are resistance training or strength training combined with aerobic training (Fatouros et al., 2002; Rogers et al., 2002; Karinkanta et al., 2007; Cancela Carral and Ayán Pérez, 2008; Sousa et al., 2013a; Lee et al., 2015; Lacroix et al., 2016; Timmons et al., 2018; Ozaki et al., 2019) on of three or more components training (Deley et al., 2007; Carvalho et al., 2010; Zhuang et al., 2014; Haripriya et al., 2018; Lichtenstein et al., 2020) and dance combined with yoga ***training*** (Im et al., 2019; Joung and Lee, 2019). At this stage, two or more combinations are called combination exercise training, but most studies are only a combination of strength or resistance training combined with aerobic exercise. Multi-components are generally three or four types of exercise. There is also a combination of aerobic and flexibility or balance components. This study focuses on aerobic exercise and reclassifies two or more combination exercise types to study the combination of exercise. The effect of physical performance is more precise and detailed, to better analyze the effect of different combinations of exercises on the elderly. Commonly, people choose a combination of strength and aerobic, while other types of composition such as balance and flexibility to combine endurance with are ignored. Balance and flexibility exercises are important because they can improve the stability of the elderly, prevent falls and relieve arthritis (Thomas et al., 2019). Trainer Cissik John pointed out that combining various training elements helps prevent loss of balance and avoid injury (Cissik, 2019).

Therefore, the goal of this study is to explore the effect of aerobic exercise combination on the physical performance among the elderly. And to explore which combination of aerobic exercise is most effective for improving the physical performance of the elderly.

## Materials and Methods

### Eligibility Criteria

We used PICOs (Population, Intervention, Comparison, Outcome and Study design) as the inclusion criteria for this review (Table 1). In addition to the above screening criteria, studies were included if (i) journal articles are full, (ii) participants healthy (not including obesity and frail) (iii) measurement of physical performance was carried out by filed objective tests. Performance is defined as physical function aspects (such as strength, endurance, flexibility, speed and agility) associated with daily activities important in maintaining independence among older adults (Guralnik et al., 1995), this study also included other important indicators which is important for elderly, such as fall, quality of life, body composition. In addition, the combination of aerobic exercise is the combination of two type of exercises, excluding the combination of exercise and other items (medication, nutrition and pure equipment which is not exercise equipment). The retrieved studies are imported into Mendeley reference management software to remove any duplicates. Firstly, the search strategies were guided by the librarian. Secondly, the title and abstract were screened by two independent reviewers BAI and XIAO. Then, relevant full text articles were selected for reading. If two reviewers disagree, the third reviewer SOH acted as the tiebreaker. Primary outcomes were performance-based measures of physical function or physical performance such as mobility, gait, muscular strength, balance, and endurance. This research has been approved by PROSPERO (ID: CRD42021213147).

**TABLE 1:** Inclusion criteria.

Items	Detail
Participants	Male and female, healthy, Age ≥ 60
Intervention	A combination of aerobic exercises, limited to combinations of exercise programs (excluding combination of diet, medicine, nutrition)
Comparison	No exercise group or exercise group in the control group
Outcome	Strength, endurance, balance, stability, agility, mobility, gait, speed, fall, flexible, quality of life, body composition
Study designs	Randomized controlled trial

### Data Sources and Search

A systematic search was carried out for articles published by 1990 or later the effects of aerobic exercise combination on physical performance in the elderly. The study was conducted and reported based on PRISMA’s statement. This review used both process and product-oriented aerobic exercise combination assessments. Four electronic databases were searched: SCOPUS, PubMed, EBSCOhost (SPORT Discus), and CINAHL Plus. The search deadline was in mid-October 2020. Keywords are citations and words that are reviewed through other people’s systematic literature (Labata-Lezaun et al., 2020). The search for items of the same aerobic exercise combination in the PubMed library was done using Mesh. Enter keywords in PubMed, the selection field option is title/abstract; title/abstract/key words on SCOPUS; Abstract on EBSCOhost (SPORT Discus) and CINAHL Plus. The keywords are as follows: (((“physical fitness” OR “functionality” OR “performance” OR “strength” OR “resistance” OR “endurance” OR “balance” OR “stability” OR “agility” OR “mobility” OR “gait” OR “speed” OR “locomotion” OR “fall” OR “handgrip” OR “SPPB” OR “tandem” OR “TUG” OR “timed up and go” OR “quality of life”) AND (“old people” OR “elders” OR “senior*” OR “old adult*” OR “aged” OR “older people” OR “older adults” OR “geriatric”) AND (“aerobic exercise* combin*” OR “acute exercise* combin*” OR “physical activity* combin*” OR “physical exercise* combin*” OR “isometric exercise* combin*” OR “exercise training* combin*”))). Terms are combined using logical operators that can be used as search tools. The authors also consulted librarians in the field.

### Study Selection

Searching for articles and deleting duplicates is done by an author BAI. The two authors independently selected the study by title and abstract. If that failed, the papers were filtered by reading the full text. Data that were extracted include: (a) author; (b) participants/age/gender; (c) Type of intervention, frequency, duration time, week and (d) main results.

### Quality Assessment

The methodological quality of the trials was assessed using the PEDro scale and to evaluate the quality of selected papers (de Morton, 2009). The PEDro scale includes 11 items designed to assess four basic methodological aspects of a study, such as randomization, blinding techniques, group comparison and data analysis processes. Trials quality assessment in the PEDro database was performed by two trained independent raters and disagreements were resolved by a third rater (Lucas et al., 2016). Item 1 (eligibility criteria) was not included in the total score because it did not affect the research’s internal validity or statistical validity. The PEDro scores ranges of 0–10 (Moseley et al., 2002). The higher the PEDro score, the higher the quality of the corresponding method. The quality of the method is evaluated using the following criteria: PEDro score of less than 5 indicates low quality, whereas a score greater than 5 indicates high quality (Maher et al., 2003; Table 2).

**TABLE 2:** Summary of methodological quality assessment scores.

References	Eligibility criteria	Random allocation	Concealed allocation	Group similar at baseline	Blind subject	Blind therapist	Blind assessor	Follow-up	Intention-to-treat analysis	Between-group comparisons	Point measure and variability	PEDro score
Fatouros et al. (2002)	1	1	0	1	0	0	0	1	0	1	1	5
Rogers et al. (2002)	1	1	0	1	0	0	0	1	0	0	1	4
Deley et al. (2007)	1	1	0	1	0	0	0	1	1	0	1	5
Karinkanta et al. (2007)	1	1	0	1	0	0	0	1	0	1	1	5
Cancela Carral and Ayán Pérez (2008)	1	1	1	1	0	0	1	0	1	1	1	7
Carvalho et al. (2010)	1	1	0	1	0	0	0	1	0	1	1	5
Sousa et al. (2013a)	1	1	0	1	0	0	0	1	0	1	0	4
Sousa et al. (2013b)	1	1	0	1	0	0	0	0	0	1	1	4
Zhuang et al. (2014)	1	1	0	0	0	0	0	1	0	1	1	4
Lee et al. (2015)	1	1	0	1	0	0	0	1	0	1	1	5
Lacroix et al. (2016)	1	0	0	1	0	0	0	1	0	1	1	4
Sousa et al. (2017)	1	1	0	1	0	0	0	1	0	1	1	5
Haripriya et al. (2018)	0	0	0	1	0	0	0	1	0	1	1	3
Timmons et al. (2018)	1	1	0	1	0	0	1	0	0	1	1	5
Im et al. (2019)	1	1	0	1	0	0	0	1	0	1	1	5
Joung and Lee (2019)	1	1	1	1	0	0	0	1	0	1	1	6
Ozaki et al. (2019)	1	1	0	1	0	0	0	1	1	1	1	6
Lichtenstein et al. (2020)	1	1	0	1	0	0	0	1	0	1	1	5
**Total**	**17**	**16**	**2**	**17**	**0**	**0**	**2**	**15**	**3**	**17**	**17**	

### Data Syntheses and Analysis

This study is Meta-aggregation of Qualitative Data Synthesis. The strength of the scientific evidence was measured by using the best evidence synthesis (BES) (Cruz-Ferreira et al., 2011). This rating system takes into account the number, methodological quality and consistency of outcomes of the studies in five levels of evidence: (1) strong evidence, provided by generally consistent findings in multiple (≥2) high quality studies, (2) moderate evidence, provided by generally consistent findings in one high-quality study and one or more low-quality studies or in multiple low-quality studies, (3) limited evidence, when only one study is available or findings are inconsistent in multiple (≥2) studies, (4) conflicting evidence, provided by conflicting findings in case–control studies (<75% of the studies reported consistent findings) and (5) no evidence, when no case–control studies are found (Burns et al., 2011).

## Results

### Study Selection

**FIGURE 1:**
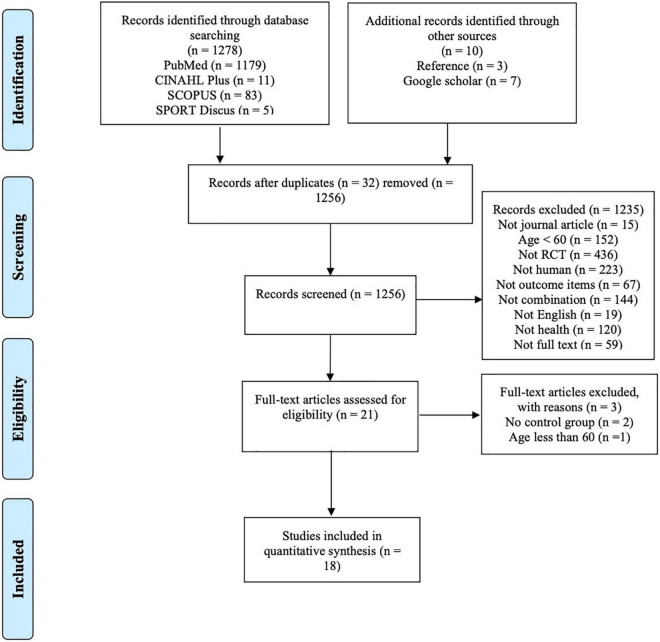
Flow chart of study selection.

The database search identified 1,278 records: SCOPUS (*n* = 83), PubMed (*n* = 1,179), EBSCOhost (SPORT Discus) (*n* = 5), and CINAHL Plus (*n* = 11). Duplicate references were deleted, and additional records were identified from other sources (*n* = 10), amounting to 1,256 articles. Through the topic and the screening, 1,235 articles were excluded leaving 21 highly relevant articles to be evaluated. After reading the full text of the 21 selected articles, 3 studies were excluded due to the lack of a control group (*n* = 2) and age less than 60 (*n* = 1). Therefore, 18 articles were selected in this literature review (Figure 1).

### Methodical Quality

The values in the PEDro scale ranged from 3 to 7 (mean = 4.83; median = 5; mode = 5). A total of 6 studies scored less than 5 while the others (*n* = 12) scored 5 or higher, indicating a mix between high-quality and low-quality studies. The publication year did not influence the quality of the studies since the low-quality studies are published in 2002 while the high-quality studies were published from 2002 to 2020 (see Table 1). The mostly met criteria were eligibility criteria (*n* = 17), group similar at baseline (*n* = 17), point measure and variability (*n* = 17), random allocation (*n* = 16), between-group comparisons (*n* = 17) and follow-up (*n* = 15). The criteria blind subject and blind therapist were not met in any of the analyzed studies, while the criterion blind assessor was met in 2 studies: concealed allocation (*n* = 2) and intention to treat analysis (*n* = 3) (Table 2).

### Characteristics of Included Studies

All study analyzed focused on physical performance and were randomized controlled trials (*n* = 18). Outcomes: 5 studies included information on Cardiorespiratory endurance, 16 had information on muscle strength, 9 had information on body composition, 5 on flexibility, 7 on fall, 7 on mobility, 8 on agility, 9 on gait, 8 items were about balance, and 2 items related to the quality of life. Populations: A total of 1,256 healthy elderly (male and female) were included in the different studies. The sample size ranged from 15 to 149 participants: men (*n* = 4), women (*n* = 5) and a mixture of men and women (*n* = 9). Age: the participants’ age ranged from 60 to 94 years old, while the average age was 65–70 years old (*n* = 10) and over 70 years old (*n* = 8). The study design included: pre-test and post-test (*n* = 11), intermediate evaluations (*n* = 4) and more than three times test (*n* = 3). Intervention: All studies used aerobic exercise combination as a research intervention. The control group was inactive in 11 studies and was active in 7 studies. A total of 11 studies combined resistance or strength training with other aerobic training methods, while the rest included multi-component training (*n* = 5), and dance-related combinations training (Korean dance and creative dance) (*n* = 2). The intervention time and frequency ranged from 4 to 52 weeks, 2 to 5 times a week and the duration of each intervention is 40–90 min with three measures of intervention intensity (repetition maximum, maximum heart rate and rating of perceived exertion) (see Table 3).

**TABLE 3:** Summary of aerobic exercise combination to improve physical performance among elderly.

References	Subject	Mean aged ± SD (range) year	Main session content (C/E)	Intervention (Wk/f/min)	Findings
Fatouros et al. (2002)	*N* = 32, M	EG1 = 70.3 ± 2.3 EG2 = 71.8 ± 2.5 EG3 = 69.8 ± -1.9 Range: 65–78	CE = NE EG = ST EG2 = CT EG3 = SA	EG1 = EG2 = EG3 = 16/3/ (45–50)	ST and SA: Isokinetic and concentric strength↑, Sit-and-reach performance↑, Elbow flexion↑, Knee flexion↑, Shoulder flexion and extension↑ and hip flexion and extension↑. CT: Hip flexion and extension↑, VO2max↑, Body weight and height ↓ isokinetic and concentric strength↑
Rogers et al. (2002)	*N* = 22, F	EG = 74.8 ± 8.8 CG = 74.7 ± 4.5 Range: 62–94	CG = NE EG = Elastic resistance bands and dumbbells	EG = 4/3/50	Elastic resistance bands and dumbbells: Grip strength↑, Balance↑, mobility↑, and agility↑,Upper and lower body flexibility↔
Deley et al. (2007)	*N* = 40, F/M, 19/21	EG = 77.2 ± 3.6	CG = NE EG = ME (walking+strength+ flexibility+Tai Chi)	EG = 52/3/60	Endurance + RES: Oxygen uptake↑, Ventilatory threshold↑, Walked in 6 min↑, Time required to cover 200 m↑,Maximal muscle strength↑.
Karinkanta et al. (2007)	*N* = 149/F	72.7 ± 2.5 Range:70–79	CG = NE EG1 = RES EG2 = Balance-jumping EG3 = RES+ balance-jumping	EG1 = EG2 = EG3 = 52/3/50	COMB: Self-rated physical functioning↑, Dynamic balance↑ RES: Leg extensor force↑ BAL: Dynamic balance↑
Cancela Carral and Ayán Pérez (2008)	*N* = 62, F	CG1 = 68.50 ± 3.4 EG2 = 68.29 ± 3.49 Range: > 65	CG1 = COM EG2 = CONS	EG1 = EG2 = 20/5/45	COM and CONS: Quality of life↑ and cognitive function↑, Balance↑ and flexibility level↑, COM: Static and dynamic strength↑.
Carvalho et al. (2010)	*N* = 65, M/F, 22/ 43	CG = 69.4 ± 4.1 EG = 68.7 ± 4.2 Range: 65–82	CG = ME EG = ME+RES	CG = 24/ 4/60 EG2 = 24/ 4/60	ME: No significant changes. CE: isokinetic muscle strength↑, knee extensors↑,DPA↔,
Sousa et al. (2013a)	*N* = 48, M	69.1 ± 5.0 Range: 65–75	CG = NE EG1 = AER EG2 = CEX	CG = EG = 32/3/55	CEX: Strength and aerobic endurance↑, body fat↓ AER: aerobic endurance↑
Sousa et al. (2013b)	*N* = 48/M	EG1 = 71.7 ± 4.7 EG2 = 68.5 ± 3.5	CG = NE EG1 = AER EG2 = CEX	EG1 = EG2 = 36/3/55	AER and CEX; Body composition↔
Zhuang et al. (2014)	*N* = 56, F/M	CG = 65.4 ± 3.97 EG = 66.3 ± 4.89 Range: 60–80	CG = NE EG = ME (walking+ strength+ flexibility+Tai Chi)	EG = 12/3/60	STB: TUG↑, 30-s chair stand↑, balance↑, strength of the extensor↑, flexor muscles at knee and ankle joints↑, walked at a faster speed with a longer step length↑, shorter support phase, and a greater sagittal plane range of motion at the hip and ankle joints↑.
Lee et al. (2015)	*N* = 19, F	EG1 = 68.38 ± 2.93 EG2 = 67.64 ± 2.82 Range: 65–75	EG1 = CEX EG2 = AER	EG2 = 8/3/60 (AER) :8/2/40 (RES) EG2 = 8/5/60	CEX: Body composition ↓ (body mass, body fat mass, percent body fat, and body mass index) isokinetic strength↑ and CVD risk factors↑. CEX > AER: Isokinetic strength↑.
Lacroix et al. (2016)	*N* = 66, F/M, 41/25	73 ± 4 Range :65–80	CG = NE EG1 = Balance+ strength (SUP) EG2 = Balance+ strength (UNSU)	EG1 = EG2 = 12/3/60	SUP and UNSU: Dynamic Steady-State Balance↔, Lower Extremity Muscle Power↑, Body Composition↔ SUP: Static steady-state balance↑, UNSU:Static steady-state balance↔.
Sousa et al. (2017)	*N* = 66, M	69.0 ± 4.9 Range: 65–79	CE = NE EG1 = AER EG2 = CEX	EG1 = 32/3/50 EG2 = 32/1/50 (RES) 32/2/50 (AER)	AER and CEX: TUG↑, Functional reach test↑, 30–s chair stand↑, 6-min walk test↑. CEX > AER: TUG↑, Functional reach test↑, 30–s chair stand↑ and 6-min walk test↑.
Haripriya et al. (2018)	*N* = 30, F/M, 15/15	72.8 ± 6.408 Range :63–91	CG = Resisted training EG = ME	CG = 10/3/40 EG = 10/3/Not clear	ME and Resisted training:8 UG↑, 6MWT↑ and SF-36↑
Timmons et al. (2018)	*N* = 84, M/F, 45/39	69.3 ± 3.5 Range: > 65	CE = NE EG1 = AER EG2 = RES EG3 = CEX	EG1 = EG2 = 36/3/40 EG3 = Half of EG1and half of EG2	RES:*C*hest press↑, Arm LBM↑, Aerobic fitness↑, Leg LBM↔ AER: Chest press↔ Arm LBM↔, Aerobic fitness↑, Leg LBM↔ CEX: Chest press↑, Arm LBM↑, Gait speed↑, Lower limb*strength*↑, Trunk fat↓, Leg LBM↔, Aerobic fitness↔
Im et al. (2019)	*N* = 25, F	71.57 ± 3.22 Range: ≥ 65	CG = NE EG = Yoga and Korean dance	EG = 12/3/60/1-6Wk (REP12), 6-12Wk (REP13)	Yoga and Korean dance: Anterior dynamic balance↑,Posterior dynamic balance↑,Static balance↑, Flexibility↑, Muscle strength↑.
Joung and Lee (2019)	*N* = 82, F/M	EG = 70.5 ± 7.89 CG = 71.77 ± 7.78 Range:65–80	EG = CD CG = STR	EG1 = EG2 = 8/2/90	STR: 30-s stand↑; 30-s arm curl↑; BBS↑, TUG↑, DGI↑and gait speed↑. CD: 30s stand↑, 30-s arm curl↑, Back stretching↑, Chair sit and reach tests↑, TUG↑, BBS↑, DGI↑ and gait speed↑
Ozaki et al. (2019)	*N* = 15, F/M, 6/9	69 ± 1 Range: > 60	CG = Walking group EG = Walking and stair-climbing	EG = (1–2 wk:3/20–25 min); (3–8 wk:3–5/30–45/ (8-17WKWS stair climbing, 25 steps, 3 days a week, 5 more steps per week.	Walking and stair-climbing: Thigh muscle size and strength↑, walking performance↑. However, stair- climbing exercise may not provide additional training effects when combined with high-intensity walking exercise.
Lichtenstein et al. (2020)	*N* = 27, M/F, 16/11	69.5 ± 5. Range:60–80	CG = TSB EG = ME	EG = CG = 8/3/50	ME and TSB: plantar flexion strength↑ and RTD↑ and trunk extension RTD↑.

*F, Female; M, male; CG, control group; NE, no exercise; EG, experimental group; AER, aerobic exercise; RES, resistance exercise; CEX, concurrent aerobic and resistance exercise; LBM, Lean body mass; TUG, Timed-Up-and-Go Test; NP, non-periodized; DUP, daily undulating periodization; SUP, supervised training; UNSU, unsupervised training unsupervised training; BAL, Balance-jumping; COMB, Combination of resistance and balance-jumping training; BMD and BMC, Bone mineral density and content; ME, Multicomponent exercise program; DPA, Daily physical activity; CD, Creative dance; STR, stretching group; BBS, Berg Balance Scale; DGI, Dynamic Gait Index; COM, combined program of aquatic exercise plus high-intensity strength training or plus calisthenic training; CONS, consisted of several aerobic, mobility and flexibility exercises; CVD, cardiovascular disease; TSB, traditional strength and balance group; RTD, rate of torque development; 6 MWT,6−MeterWalkingTest;; 8 UG, 8-Up-and-Go; ST, strength training; CT, cardiovascular training; SA, combination of strength and aerobic training. There was a significant increase before and after exercise training, ↓significant decrease before and after exercise training↑, There was no significant change in training before and after exercise.↔, xercise combine with exercise +.*

### The Impact of Combination of Aerobic Exercise on Physical Performance

The research results were measured based on the study population, time of intervention, means of intervention, and the impact of the results.

#### Aerobic and Strength/Resistance Training on Physical Performance

There are 16 studies on strength (Fatouros et al., 2002; Rogers et al., 2002; Deley et al., 2007; Karinkanta et al., 2007; Cancela Carral and Ayán Pérez, 2008; Carvalho et al., 2010; Sousa et al., 2013a,b, 2017; Zhuang et al., 2014; Lee et al., 2015; Timmons et al., 2018; Im et al., 2019; Joung and Lee, 2019; Ozaki et al., 2019; Lichtenstein et al., 2020). Grip strength was measured using Hydraulic hand dynamo meter (Rogers et al., 2002; Cancela Carral and Ayán Pérez, 2008; Timmons et al., 2018). The comparison between pre-test and post-test showed that the effect of exercise on grip strength was significant in the combination group (Rogers et al., 2002; Cancela Carral and Ayán Pérez, 2008; Timmons et al., 2018) training methods include a combination of aerobic and resistance exercise (CEX) (moderate evidence) (Rogers et al., 2002; Timmons et al., 2018) exercising in water combine with strength training (limited evidence) (Cancela Carral and Ayán Pérez, 2008). However, the impact of the exercises can be summarized as the following: large aerobic training (AER) < resistance training (RES) < CEX (limited evidence) (Timmons et al., 2018). The training population includes women and a mix of men and women. Lower limb strength was measured as follows: 1 repetition maximum (1 RM) on leg press (Fatouros et al., 2002; Timmons et al., 2018) 1 RM (Deley et al., 2007; Sousa et al., 2017), 30-s chair stand test (Rogers et al., 2002; Sousa et al., 2013a,^2017^; Zhuang et al., 2014; Im et al., 2019; Joung and Lee, 2019), 6-min walk test (Sousa et al., 2017), and both knees were measured by Custom-made isokinetic dynamometer (Carvalho et al., 2010). Isokinetic strength and knee joint strength were measured by Cybex NORM isokinetic system (Lee et al., 2015; Ozaki et al., 2019). Maximum extensive strength of the legs was measured by dynamometric platform (Cancela Carral and Ayán Pérez, 2008).

There was a significant main effect between time × group interaction in lower limb strength for CEX (Fatouros et al., 2002; Sousa et al., 2013a,^2017^) combination of yoga and Korean dance training (Im et al., 2019) creative dance training (Joung and Lee, 2019). There was also a significant main effect of time in lower limb strength for combination of ME (strong evidence) (Deley et al., 2007; Carvalho et al., 2010; Zhuang et al., 2014), walking and stair-climbing (Ozaki et al., 2019), exercising in water combined with strength training (Cancela Carral and Ayán Pérez, 2008). A single exercise program can also improve lower limb strength such as elastic band and dumbbells (Rogers et al., 2002) and CEX (strong evidence) (Sousa et al., 2013a,^2017^; Lee et al., 2015; Timmons et al., 2018). In addition, the impact goes from large to small CEX (*p* < 0.001) > AER (*p* < 0.001) > RES (*p* < 0.001) (limited evidence) (Timmons et al., 2018) and strength training (*p* < 0.05) > strength combined with cardiovascular training (*p* < 0.05) > cardiovascular training (*p* < 0.05) (limited evidence) (Fatouros et al., 2002). In contrast, some of these exercises did not have a significant effect on lower limb strength such as ME (limited evidence) (Carvalho et al., 2010), and AER (walking) (moderate evidence) (Sousa et al., 2013a; Lee et al., 2015). Upper limb strength was measured as follows: chest press machines (Timmons et al., 2018), arm curl test (number of repetitions within the 30s (Rogers et al., 2002; Joung and Lee, 2019) and 1RM test (Fatouros et al., 2002) limb strength for creative dance (Joung and Lee, 2019), a combination of strength and AER (Fatouros et al., 2002) and ME (Lichtenstein et al., 2020). Moreover, there was a significant effect of time in upper limb strength for CEX (limited evidence) (Timmons et al., 2018) and a combination of elastic band and dumbbell training (limited evidence) (Rogers et al., 2002).

#### Cardiorespiratory Endurance

There were five studies that assessed endurance among the elderly (Deley et al., 2007; Sousa et al., 2013a; Haripriya et al., 2018; Timmons et al., 2018; Lichtenstein et al., 2020). Cardiopulmonary function evaluation index includes step test (Timmons et al., 2018), Six-Minute Walk Test (6 MWT) and walking 2,000 m as fast as possible (Haripriya et al., 2018; Lichtenstein et al., 2020) electromagnetically braked cycle ergo-meter and 6-min and 200-m walk tests (moderate evidence) (Deley et al., 2007; Sousa et al., 2013a). There was a significant effect between time × group interaction in endurance for strength and aerobic endurance training (limited evidence) (Sousa et al., 2013a), traditional strength combined with balance training and ME (limited evidence) (Lichtenstein et al., 2020). There was also a significant effect of time in endurance for CEX (limited evidence) (Timmons et al., 2018), ME (limited evidence) (Deley et al., 2007; Haripriya et al., 2018). However, aerobic fitness was improved at post-test in AER and RES, but not at either time point in CEX (limited evidence) (Timmons et al., 2018). The study population includes men (1 study) and mixed men and women (4 studies).

#### Balance

There were eight studies related to balance (Rogers et al., 2002; Karinkanta et al., 2007; Cancela Carral and Ayán Pérez, 2008; Zhuang et al., 2014; Lacroix et al., 2016; Im et al., 2019; Joung and Lee, 2019; Lichtenstein et al., 2020) including static balance, dynamic balance, and functional balance. Static balance was measured by Kistler force platform (Lichtenstein et al., 2020), flamingo test (Cancela Carral and Ayán Pérez, 2008), and modified Romberg Test (Lacroix et al., 2016). Meanwhile, dynamic balance was measured using the functional reach test (FR), the star excursion balance tests (SEBTs) (Zhuang et al., 2014) and walking on a 10-meter walkway (the OptoGait System) (Lacroix et al., 2016). Static and dynamic balance were determined through the single-leg eyes open stand, Y-balance test, and reactive balance (Lichtenstein et al., 2020), and Center of Pressure measuring method of the Humac Norm Balance System (Im et al., 2019). Apart from that, functional balance was measured by the Berg Balance Scale (BBS) (Joung and Lee, 2019). Dynamic balance and agility were measured by standardized figure-of-8 running test around two poles placed 10 m apart Self-rated (Karinkanta et al., 2007). Finally, balance, agility, and mobility were measured by Up-&-Go Test (Rogers et al., 2002).

A significant group × time interaction was found in static balance for creative dance group, but not stretching group (limited evidence) (Joung and Lee, 2019), and Korean dance and training (limited evidence) (Im et al., 2019). There was also a significant effect of time in static balance for combined program of aquatic exercise plus high-intensity strength training that consisted of several aerobic, mobility and flexibility training (limited evidence) (Cancela Carral and Ayán Pérez, 2008), strength and balance training and the 8-form Tai Chi Chuan ***training*** (limited evidence) (Zhuang et al., 2014), and combination of bands and dumbbell training (limited evidence) (Rogers et al., 2002). Dynamic balance improved in the balance-jumping training and the combination of resistance and balance-jumping training (limited evidence) (Karinkanta et al., 2007).

There is no significant difference for between-group in balance for ME and traditional strength and balance training (limited evidence) (Lichtenstein et al., 2020). There is no significant difference of time in balance for combination of balance and strength training (limited evidence) (Lacroix et al., 2016). The study population includes women (4 studies) and mixed men and women (4 studies).

#### Fall, Gait, Mobility, and Agility

One study evaluated fall risk using the Falls Efficacy Scale International (FES-I). Dynamic balance and agility were assessed with a standardized figure-of-8 running test around two poles placed 10 m apart (Karinkanta et al., 2007). There are 3 ways to measure gait: Functional gait performance was assessed with Dynamic Gait Index (DGI) (Joung and Lee, 2019); Gait speed was assessed with 10-Meter Walking Test (10 MWT) (Joung and Lee, 2019); eight-camera VICON motion analysis system (Zhuang et al., 2014).

The other 6 articles used TUG to measure mobility, fall risk, gait, and agility (Rogers et al., 2002; Zhuang et al., 2014; Sousa et al., 2017; Haripriya et al., 2018; Timmons et al., 2018; Joung and Lee, 2019). All the studies here show that the effects of training on TUG in older adults are significant. A significant group × time interaction was found in TUG for CEX (limited evidence) (Sousa et al., 2017), creative dance training (limited evidence) (Joung and Lee, 2019). There was a significant difference for time in TUG for CEX (limited evidence) (Timmons et al., 2018), combination of bands and dumbbell training (limited evidence) (Rogers et al., 2002), combination of resistance and balance-jumping training (limited evidence) (Karinkanta et al., 2007), ME (limited evidence) (Haripriya et al., 2018) and core strength and balance training and the 8-form Tai Chi Chuan ***training*** (limited evidence) (Zhuang et al., 2014). The study population includes women or men (1 studies) and mixed men and women (4 studies).

#### Flexibility

There were five studies related to flexibility consisting of upper limb flexibility and lower limb flexibility (Fatouros et al., 2002; Rogers et al., 2002; Cancela Carral and Ayán Pérez, 2008; Im et al., 2019; Joung and Lee, 2019). Lower-body flexibility was measured by sit and reach test (Fatouros et al., 2002; Rogers et al., 2002; Cancela Carral and Ayán Pérez, 2008; Im et al., 2019; Joung and Lee, 2019) and goniometer as described by Norkin and White (Fatouros et al., 2002). Upper-body flexibility was measured by a scratch test (Rogers et al., 2002; Joung and Lee, 2019) and goniometer as described by Norkin and White (Fatouros et al., 2002).

There was also a significant effect for time in lower-body flexibility in the combined aquatic exercise plus high-intensity strength training and a combination of several aerobic (CHC), mobility and flexibility exercise (limited evidence) (Cancela Carral and Ayán Pérez, 2008). There were significant effects between time × group interaction in lower-body flexibility creative dance training and stretching training, and Korean dance combined with yoga training (limited evidence) (Im et al., 2019). However, there were no changes observed when strength training was combined with AER (limited evidence) (Fatouros et al., 2002).

A significant group × time interaction was found in back stretching for the creative dance group, but not for stretching training (limited evidence) (Joung and Lee, 2019), while another study did not show any change in flexibility (limited evidence) (Rogers et al., 2002). The study population consisted of women (1 study) and mixed men and women (1 study).

#### Body Composition

There were 9 studies related to body composition (Rogers et al., 2002; Carvalho et al., 2010; Sousa et al., 2013a,b, 2017; Lee et al., 2015; Lacroix et al., 2016; Timmons et al., 2018; Im et al., 2019). Nonetheless, no significant changes were observed in relation to body composition (strong evidence) (Rogers et al., 2002; Carvalho et al., 2010; Sousa et al., 2013a,^2017^; Lacroix et al., 2016; Timmons et al., 2018; Im et al., 2019). The evaluation methods used in these studies include elastic resistance bands and dumbbells ***training*** (limited evidence) (Rogers et al., 2002), combined ME plus resistance training (limited evidence) (Carvalho et al., 2010), CEX (Moderate evidence) (Sousa et al., 2013a,^2017^; Timmons et al., 2018), balance and strength training (limited evidence) (Lacroix et al., 2016), and yoga and Korean dance training (limited evidence) (Im et al., 2019). Some of these exercises contributed to the significant decrease in body composition criteria like body mass, body fat mass, body fat percentage, and body mass index whereas lean mass was increased significantly in the CEX group compared with those in the AER group on elderly women (Lee et al., 2015).

The training time is less than or equal to 12 weeks. There is only 1 article with an intervention time greater than 36 weeks, and body composition changes are seen in the resistance training group.

#### Other Outcomes

A total of 2 studies assessed participants’ quality of life (Cancela Carral and Ayán Pérez, 2008) *via* Health Orientation Scale (Cancela Carral and Ayán Pérez, 2008) and SF-36 (Haripriya et al., 2018). Based on the results obtained, combination exercise appeared to significantly impact the quality of life (Cancela Carral and Ayán Pérez, 2008; Haripriya et al., 2018). The training methods used were combined programs of aquatic exercise plus high-intensity strength training or plus calisthenic training (Cancela Carral and Ayán Pérez, 2008) and ME (Haripriya et al., 2018). The study population consisted of women (1 study) and mixed men and women (1 study).

One study evaluated the aspect of power. Lower extremity muscle power was assessed with Chair Stand Test and Stair Ascent and Descent Test (SADT) (Lacroix et al., 2016). After 12 weeks of training, there was a significant difference in power for balance combined with strength training on the elderly (Lacroix et al., 2016).

#### Outcomes of Comparison Between Single and Combination Group

Some studies have shown that combined training has a more comprehensive effect on the physical performance of the elderly than a single aerobic training (strong evidence) (Fatouros et al., 2002; Cancela Carral and Ayán Pérez, 2008; Carvalho et al., 2010; Sousa et al., 2013a,b, 2017; Lee et al., 2015; Timmons et al., 2018; Lichtenstein et al., 2020). The CEX has additional effects compared to AER and RES in terms of gait speed, lower limb strength and trunk fat (limited evidence) (Timmons et al., 2018). Meanwhile, CEX was more impactful than AER (strong evidence) (Rogers et al., 2002; Deley et al., 2007; Zhuang et al., 2014; Lee et al., 2015; Lacroix et al., 2016; Haripriya et al., 2018; Jia et al., 2018; Timmons et al., 2018) in the TUG, functional reach test,30-s chair standand 6-min walk test (Sousa et al., 2017). The combination of resistance and balance-jumping training has additional benefits than balance jumping and RES such as in self-rated physical functioning (limited evidence) (Karinkanta et al., 2007).Furthermore, the ME+RES has improved isokinetic muscle strength and knee extensors better than ME (limited evidence) (Carvalho et al., 2010). Moreover, the strength + AER resulted in better outcomes than AER in terms of sit-and-reach performance, elbow flexion, knee flexion, shoulder flexion and extension (limited evidence) (Fatouros et al., 2002). Additionally, CEX has improved strength and body fat percentage better than AER. There was no significant difference between walking and stair-climbing group and walking group (limited evidence) (Ozaki et al., 2019).

## Discussion

NCDs are by far the leading cause of death in the world, accounting for 63% of all deaths (World Health Organization, 2020). In addition, falls have become the second leading cause of accidental injuries among the elderly, except for traffic accidents. However, in the case of multiple health threats, a single type of exercise cannot prevent and protect the multiple health threats for the elderly. Therefore, this article explores the effects of multiple exercise combinations on physical performance, preventing certain diseases, falls and injuries, and provides exercise programs for the elderly to improve physical fitness and prevent certain diseases.

The main aerobic combination training generally involves the combination of two or more training components (Rogers et al., 2002; Deley et al., 2007; Zhuang et al., 2014; Lee et al., 2015; Lacroix et al., 2016; Haripriya et al., 2018; Jia et al., 2018; Timmons et al., 2018). Some studies include two exercise components, and some involve more than two. Different combinations have different effects on the elderly. The three main aerobic exercises according to the frequency in research articles used in this study are detailed in Table 4.

**TABLE 4:** Ranking of interventions based on numbers of studies.

Intervention type	# of studies	Percentage
Resistance/strength+aerobic exercise walking/dancing/jogging/step aerobics/dumbbells/exercising in water/climbing stairs/cross trainer)	11	61.11%
Combination of multi-components	5	27.78%
Dancing+ yoga/creative multimodal	2	11.11%

All three aerobic exercise combinations have a significant impact on physical performance indicators in older adults, but since the specific training methods used in each study are different, the evidence for the results of most studies is limited, it is impossible to determine which aerobic combination intervention is best or which parameter has the greatest impact on physical performance indicators. Regarding the effect of aerobic combination training on the physical performance of the elderly, most components of physical performance still need more research to prove, except for the ME combination and CEX can improve lower limb strength and no significant changes were observed in relation to body composition. However, through the analysis of the previous literature, more appropriate and safe exercise methods can be recommended for the elderly.

The main aerobic combination training are: resistance training or strength training (there are three types of training: equipment, body weight and climbing stairs) combined with AER (Fatouros et al., 2002; Rogers et al., 2002; Karinkanta et al., 2007; Cancela Carral and Ayán Pérez, 2008; Sousa et al., 2013a,b, 2017; Lee et al., 2015; Lacroix et al., 2016; Timmons et al., 2018; Ozaki et al., 2019) multi-component/multi-elements training (Deley et al., 2007; Carvalho et al., 2010; Zhuang et al., 2014; Haripriya et al., 2018; Lichtenstein et al., 2020) and Korea dance combined with yoga/creative multielement) (Im et al., 2019; Joung and Lee, 2019). Three training methods have significant positive effects on older adults’ physical performance parameters such as strength (upper and lower body strength), dynamic balance, fall risk, mobility, gait, agility, flexibility. However, none of these three training methods was proven significant in improving parts of body composition in elderly women in all the studies used in this paper except for 1 article (Lee et al., 2015). Meanwhile, another three studies reported that CEX training did not significantly affect the body composition of older men (Sousa et al., 2013a,b, 2017). With the increase in age, men’s weight decreases gradually, whereas women’s weight gradually increases (JafariNasabian et al., 2017). Therefore, CEX (Lee et al., 2015) changes the body composition of women more significantly than men.

In addition, aerobic (cross trainer and stationary cycle) combined resistance training had no impact on aerobic function for the elderly (Timmons et al., 2018). Aerobic adaptation stimulus provided by the two exercise modes alone needs to exceed thresholds not provided by CEX in the respective modes (Taaffe et al., 1995; Sundstrup et al., 2016). Elastic resistance bands and dumbbells training could not change flexibility performance (Rogers et al., 2002). Earlier data has shown that yoga, Pilates, dance, Tai Chi and stretching can improve the flexibility of the elderly (Saravanakumar et al., 2018). On the contrary, multi-component training of 50 min, three times a week for eight weeks did not affect static balance (Lichtenstein et al., 2020). Balance training is considered task specific (Kümmel et al., 2016) and programs to improve balance suggest 90–120 min of balance training per week for 11–12 weeks (Lesinski et al., 2015). In contrast, two times a week, 60 min of multi-component training did not significantly affect lower limb strength (Carvalho et al., 2010). The ACSM guidelines recommend at least 150 min of moderate-intensity aerobic exercise or 75 min of Vigo rate-intensity aerobic exercise, and older adults will reap significant benefits (Lee et al., 2017).

A total of 18 studies reported that strength, body composition, and gait accounted for 50% or more of the total strength in their research (see Table 5), while the rest were below 50%. Meanwhile, fewer than 30% of studies have reported the impact of ***combination exercise training*** on quality of life, endurance, and flexibility among older adults. This observation suggests that studies regarding aerobic combination training on physical performance in the elderly are still lacking. Moreover, there were very few studies comparing single training and combined training. Most of these combination exercises training had a greater effect on physical performance in older adults than single exercise training (Fatouros et al., 2002; Karinkanta et al., 2007; Cancela Carral and Ayán Pérez, 2008; Carvalho et al., 2010; Sousa et al., 2013a,b, 2017; Lee et al., 2015; Haripriya et al., 2018; Timmons et al., 2018; Lichtenstein et al., 2020). This was because the combination exercises involve more elements that engage in physical performance, so they produce more comprehensive and effective results than single exercises (U.S. Department of Health and Human Services, 2021).

**TABLE 5:** Percentage of the total number of studies that involved physical indicators.

Outcomes	# of studies	Percentage
Cardiorespiratory endurance	5	27.78%
Strength	16	88.89%
Body composition	9	50.00%
Flexibility	5	27.78%
Fall and mobility	7	38.89%
Agility	8	44.44%
Balance	8	44.44%
Quality of life	2	11.11%
Gait	9	50.00%

CEX (Sousa et al., 2013a,b, 2017; Cadore et al., 2014) and ME (Cadore et al., 2013; Echeverria et al., 2020; Kim et al., 2020) appeared to be the most effective interventions for improving the overall health of frail older adults. It is not difficult to show that changes in physical performance also have a positive effect on the health of the elderly. This study found that compared with the lack of changes in AER and RES, CEX has a significant effect in reducing trunk fat and abdominal fat (Timmons et al., 2018) compared with AER, CEX is more effective in improving falls and cardiovascular disease risk factors (Fatouros et al., 2002; Sousa et al., 2013a; Lee et al., 2015), especially in changing blood pressure and blood lipids (Sousa et al., 2013b) such as balance, posture control, mobility and leg strength (Sousa et al., 2017) CEX also positively reflects a significant reduction in overall risk factors for the elderly (Sousa et al., 2013b). Strength and water exercise can help elderly women who exercise regularly participate in high-frequency and high-intensity training programs. While not affecting their health, their quality of life, cognitive function, independence and physical health are improved (Cancela Carral and Ayán Pérez, 2008). However, the combination of stair climbing exercise and high-intensity walking exercise may not produce additional training effects (Ozaki et al., 2019). In general, ME group compared with the non-exercise group, even under low-intensity conditions, multi-component long-term training for healthy subjects over 70 years old can significantly improve their maximum strength, exercise capacity and function of the lower limbs (Deley et al., 2007). The 12-week ME program improved physical performance and gait parameters related to falls (Zhuang et al., 2014; Haripriya et al., 2018). However, ME is effective for the elderly. The effect of muscle strength is small (Carvalho et al., 2010). Current research results of dance and yoga combined with creative multi-model dance exercise show that the two are effective in improving strength, flexibility, functional balance and mobility (Im et al., 2019; Joung and Lee, 2019). DHEA-S and the level of estrogen has a significant effect (Im et al., 2019). These research results provide a basis for a comprehensive exercise program as a new exercise strategy for women’s healthy aging (Im et al., 2019; Joung and Lee, 2019).

Future research should explore the impact of different combination exercises on the elderly and provide targeted combination exercise programs for the elderly suffering from different types of diseases. At present, CEX and ME have been widely studied, and this research has also been shown to have a positive effect on the physical performance and disease relief of the elderly. However, research on combinations of exercises is still rare, and other types of groups and exercises are ignored, such as a combination of endurance and balance exercise components, combination of flexibility and balance exercise components. And the evidence for the effects of various combination exercises on the body composition of the elderly is limited. Therefore, the effect of combined exercise on the physical performance of the elderly still needs more research to be proven. The consistency of aerobic endurance training is vital to maintaining a healthy body, but it has been found to be challenging for most elderly people to persist (Jancey et al., 2007; Hawley, 2009; Nyman et al., 2011; Ashdown-Franks et al., 2020). Therefore, a creative combination of two methods or multiple exercise components needs to be established to provide more benefits and attract more elderly people.

### Study Limitation

In this study, four databases be included which was SCOPUS, PubMed, EBSCOhost, and CINAHL Plus to find 18 articles. Thus, there is enough studies to support the main points of research in spite of no more databases are included. Only 2 studies (Karinkanta et al., 2007; Joung and Lee, 2019) have a sample size of more than 30 people per group. Besides, none of the articles included the sample size calculations to advance the rigor of the experiment. The sample age range is relatively large; only 3 studies have an age range of less than or equal to 10 years old (Karinkanta et al., 2007; Sousa et al., 2013a; Lee et al., 2015). Relatively few studies only study men (Fatouros et al., 2002; Sousa et al., 2013a,b, 2017) or women (Rogers et al., 2002; Karinkanta et al., 2007; Cancela Carral and Ayán Pérez, 2008; Lee et al., 2015; Im et al., 2019). Secondly, the means and frequency of intervention are the main factors affecting intervention results. The intervention time often ranges between 4 weeks and 52 weeks, while 5 studies (Rogers et al., 2002; Lee et al., 2015; Haripriya et al., 2018; Joung and Lee, 2019; Lichtenstein et al., 2020) were conducted within 10 weeks or less. The frequency of 2 studies interventions was twice a week. The rest of the studies conducted their experiments within three or more weeks. The duration of each intervention is between 40 min (Timmons et al., 2018) and 90 min (Joung and Lee, 2019).

Most papers studied partly physical performance index. One of the studies showed a comprehensive physical performance (Joung and Lee, 2019). Subsequently, it’s impossible to know which exercise has a more comprehensive effect on physical performance in older adults.

There are 3 studies (Fatouros et al., 2002; Karinkanta et al., 2007; Timmons et al., 2018) include a control group, single exercise group and a combination of exercise group.

Future studies should have a larger sample size, or a sample size calculated scientifically given the limitations highlighted. Apart from that, men and women should be studied independently, and the age range of participants should not be too extensive. Besides, the total duration of the intervention and the duration of each intervention should not be too short. Future research needs to cover the control group, each single group and combination group. Researchers rarely explore the combination of multiple forms of exercise.

## Conclusion

There is strong evidence to prove that aerobic combined training has a more comprehensive and practical effect on the physical performance of the elderly than AER alone. However, evidence for the effect of most combination exercises on the physical performance of the elderly is still lacking. ACSM’s Exercise for Older Adults point out: Elderly should combine multiple sports with exercising their physical strength, endurance, flexibility, and balance (Chodzko-Zajko, 2014). However, most of the research in this review involves a combination of strength/resistance and aerobic exercise. National Institute on Aging (NCOAging, 2021) has shown that diversified exercises help reduce the elderly’s boredom and the risk of injury and at the same time improve their balance and flexibility which were essential to protect the stability of the elderly and prevent falls but commonly neglected in the exercise program carried out by the elderly (U.S. Department of Health and Human Services, 2021). Therefore, there should be a combination of multiple components, more than two or all. It will promote the overall improvement of the physical fitness of the elderly and prevent the body from losing balance and injury due to the improvement of part of the physical fitness (Cissik, 2019).

## Data Availability Statement

The original contributions presented in the study are included in the article/supplementary material, further inquiries can be directed to the corresponding author/s.

## Author Contributions

XB and WX performed the literature search, selection of studies, and study quality assessment. Following an initial screen of titles and abstracts (XB and WX), full scrutiny of potentially eligible studies was independently screened by XB and WX using the specific inclusion criteria. KGS arbitrated any disagreements in study inclusion. All authors contributed to manuscript revision, read, and approved the submitted version.

## Conflict of Interest

The authors declare that the research was conducted in the absence of any commercial or financial relationships that could be construed as a potential conflict of interest.

## Publisher’s Note

All claims expressed in this article are solely those of the authors and do not necessarily represent those of their affiliated organizations, or those of the publisher, the editors and the reviewers. Any product that may be evaluated in this article, or claim that may be made by its manufacturer, is not guaranteed or endorsed by the publisher.
